# Prevalence of Mouse Mammary Tumor Virus (MMTV)-like sequences in human breast cancer tissues and adjacent normal breast tissues in Saudi Arabia

**DOI:** 10.1186/s12885-018-4074-6

**Published:** 2018-02-09

**Authors:** Reem Al Dossary, Khaled R. Alkharsah, Haitham Kussaibi

**Affiliations:** 1 Department of Microbiology, Collage of Medicine, Imam Abdulrahman Bin Faisal University (IAU), 1982, Dammam, 31441 Kingdom of Saudi Arabia; 2 Department of Epidemic Diseases Research, Institute for Research and Medical Consultation, Imam Abdulrahman Bin Faisal University, Dammam, 31441 Saudi Arabia; 3 Department of Pathology, King Fahd Hospital of the University, Imam Abdulrahman Bin Faisal University, Dammam, 31441 Saudi Arabia

**Keywords:** Mouse mammary tumor virus, Human breast cancer, Prevalence

## Abstract

**Background:**

Breast cancer is considered the most common cancer in women worldwide and is the leading cause of cancer mortality. Sequences similar to Mouse Mammary Tumor Virus (MMTV) were detected in human breast cancer in several studies from different geographical areas. However, the role played by this virus in breast cancer tumorigenesis is not completely understood. These MMTV-like sequences were found to be associated with breast cancer of more malignant types. The aim of this study is to determine the prevalence of MMTV-like envelope gene (*env)* positivity in breast cancer and non-cancerous breast tissue from Saudi Arabia.

**Methods:**

Detection of MMTV-like *env* proviral sequences was done using newly designed primers for conventional polymerase chain reaction (PCR). One hundred nighty four samples were collected from 103 females with breast cancer in addition to 51 control breast tissue obtained from individuals without cancer. We additionally investigated the association of proviral positivity with age of the patients, grade of breast cancer and presence of lymph node metastasis. The results were confirmed by sequencing.

**Results:**

The prevalence of MMTV-like *env* proviral positivity was 8.7% (9/103). MMTV *env* proviral sequences were detected in 5.9% (6/101) of breast cancer tissues and 9.7% (9/93) of non-cancerous adjacent tissues obtained from the same patients. None of the 51 control sample showed positive result for the MMTV *env* gene. No significant association was found between detection of the virus and the age of the patient, grade of the cancer or presence of metastasis.

**Conclusion:**

We document the presence of low frequency of MMTV *env* provirus sequence among breast cancer patients from Saudi Arabia. Further studies are needed to explore the role of the MMTV in breast cancer.

## Background

Breast cancer is considered the leading cause of cancer morbidity and mortality worldwide accounting for 23% of cancer in females and 14% of cancer mortality [1] with 40–80% five year survival rate [2].

The association between human breast cancer and viruses can be dated back to the 1971, at the University of Columbia, New York, when they first demonstrated the presence of viral particles in human milk, by electron microscopy, which was found to be similar to Mouse Mammary tumor virus (MMTV) the causative agent of mammary tumor in mice [3]. In 1972, Axel et al. was able to detect viral particles in 79% (30/38) of breast cancer samples and these viral particles were found to have RNA related to Mouse Mammary Tumor Virus (MMTV) by hybridization and also carry an RNA-dependent DNA polymerase (reverse transcriptase) [4]. The virus was not detected in 10 control samples.

Being a retrovirus, detection of MMTV-like proviral sequences in the pathological human samples was markedly confounded with Human Endogenous Retroviruses (HERV) which constitutes around 8% of the human genome. With the improvement of molecular techniques, namely sequencing, detection of MMTV-like sequences was achieved using highly specific primer pairs that detect envelope gene (*env*) sequences of MMTV with low homology to HERV [5]. MMTV-like *env* sequences were detected in 38–40% (121/314) of human breast cancer samples, 6.9% (2/29) of fibroadenomas and 1.8% (2/107) normal breast tissues but not in other tumor tissues and normal tissues obtained from the same patients [5]. MMTV–like 250 bp *env* sequences was also detected in 30.1% breast cancer samples but not from normal breast samples obtained from the same patient indicating exogenous origin of the virus [6]. MMTV-like *env* sequences was detected in breast cancer and lymphoma specimen from the same patient [7]. Interestingly, MMTV like DNA sequences with 98–99.6% homology to MMTV *env* sequences were detected in a cluster of father, mother and a daughter with breast cancer in Canada indicating possible common source of transmission [8]. In addition, MMTV was detected in the nucleus of human breast cancer specimen using in situ PCR [9, 10].

Furthermore, expression of MMTV sequences was detected in 66% of DNA positive samples [11, 12] and MMTV protein was also detected in human breast cancer cell line T47D [13] and human breast tissues [14] indicating that active expression of the virus and its proteins might be involved in the pathogenesis of human breast cancer since the majority of DNA positive sample shows evidence of viral expression and protein synthesis.

Human mammary carcinoma cell line T47D was found to produce retroviral particle of B type morphology (eccentric dense core) which hybridize with MMTV DNA under stringent conditions and show immune reactivity with anti-MMTV antibodies [15]. To overcome the difficulty of acquiring a conclusion from an established cell line with possible contamination, primary culture of human breast cancer was developed and showed production of viral particles of B type morphology [16].

Complete structure of MMTV- like provirus, also known as Human Mammary Tumor Virus (HMTV), was described as 9.9 kb that show 95% and 57% homology with MMTV and HERV-K10 respectively. Homology to HERV-K10 was mainly in 3.5 kb region of *gag* and *pol* genes [16].

Interestingly, MMTV-like *env* sequence positivity was associated with more malignant types of breast cancers namely gestational and inflammatory breast cancer [17, 18].

Taking in consideration that MMTV-like sequence was mainly detected in breast cancers of advanced stage with the fact that breast cancer in Saudi women, particularly those of the Eastern Province, has an earlier age of onset and worse prognosis [19], we sought to determine the prevalence of MMTV positivity among Saudi breast cancer patients and correlate it with the observed pattern of the disease.

## Methods

### Study design and samples

**Fig. 1:**
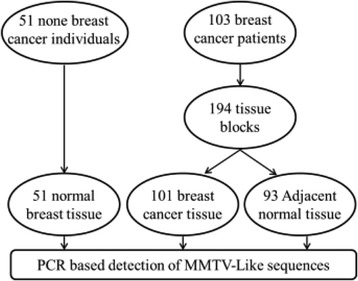
Schematic representation of the samples and study population

A retrospective study was done to determine the prevalence of MMTV-like *env* proviral sequences in breast cancer tissues in the eastern province of Saudi Arabia. A total of 103 females with breast cancers were included and a total of 194 formalin fixed paraffin embedded (FFPE) samples were tested (Fig. 1). Sixty seven females were Saudi and 36 were non Saudi females living in Saudi Arabia. The samples were collected at king Fahd Hospital of the University in Al Khobar, Saudi Arabia, in the period between 2009 and 2014. Out of 194 samples, 101 samples where breast cancer tissues and 93 samples were non-cancerous normal breast tissues obtained from non-cancerous surrounding tissues of the same patients (safety margins) (Fig. 1). Additionally, 51 FFPE breast tissues from non-breast cancer patients were collected from the hospital repository. These tissue biopsies were collected either for reduction mammoplasty (5 cases) or from patient complaining of a breast mass to rule out malignancy. Twenty six of these biopsies turned to be fibroadenomas, seventeen biopsies were fibrocystic changes, two biopsies were diagnosed as duct ectasia, and one biopsy was granulomatous mastitis.

Ethical approval for this study was obtained from the Standard Committee for Research Ethics on Living Creatures (SCRELC) at Imam Abdulrahman Bin Faisal University (IRB approval number IRB-2013-01-006).

### Tissue preparation for extraction:

Two 10 μm sections were cut from each FFPE tissue block. A scalpel was used to trim the excess paraffin around the tissue. The tissue section was then immediately deparaffinized using deparaffinization solution (Qiagen, Hilden, Germany) according to the manufacturer’s instructions. To avoid cross contamination, each sample was processed separately and the microtome blade was brushed and cleaned with alcohol before each use.

### DNA and RNA extraction

Extraction of DNA and RNA was done using Allprep DNA/RNA FFPE kit (Qiagen, Hilden, Germany) according to the manufacturer’s instructions. The kit allows simultaneous extraction of DNA and RNA in separate tubes. Quantity and quality of extracted nucleic acid was determined using the NanoDrop 2000 (Thermo Fisher Scientific, Massachusetts, United States). The extracted nucleic acid was stored at − 70 °C till the time of analysis.

### Polymerase chain reaction

**Fig. 2:**
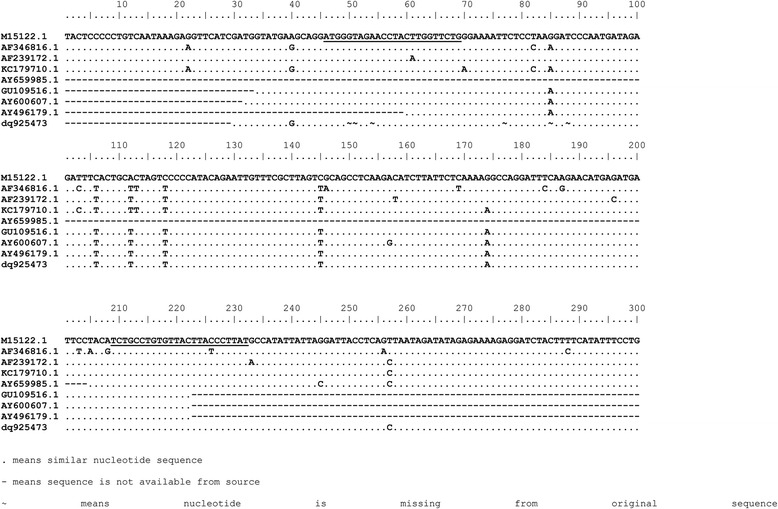
Alignment of MMTV and HTMV *env* sequences. The position of primers used in the study is underlined

A fragment from the β-globin gene was amplified using the following primers beta-globin-F 5-TAAGGTGAAGGCTCATGGCAA-3 and beta-globin-R 5-GCAGCTCACTCAGTGTGGCA-3 and the following conditions 95 °C for 5 min, and 35 cycles of 95 °C for 30 s, 58 °C for 30 s, and 72 °C for 25 s [20]. For the detection of MMTV-*env* like sequences, a set of newly designed primers based on the alignment in Fig. 2 (MMTV-F 5’-ATGGGTAGAACCTACWTGGTTCTG-3’and MMTV-R 5’-ATAAGGRTAAGTAACACAGGCAGA-3′) were used according to the following conditions: 94 °C for 5 min, followed by 35 cycles of 94 °C for 30 s, 53 °C for 30 s, and 72 °C for 25 s. Flexi taq polymerase (promega) was used with 2.5 mM MgCl_2_ per reaction. Negative, no template control, was included in each run. Positive control (PCR fragment of MMTV *env* gene kindly provided by Prof. Pogo) was also included in each run. To exclude any possibility of contamination, we have strictly worked in discrete areas of pre and post PCR and the positive control was added separately in another room.

### Reverse transcriptase PCR (RT-PCR)

RNA fraction from the Allprep DNA/RNA FFPE kit was used for RNA extraction. DNase digestion step was performed additionally during the extraction of RNA. The Sensiscript reverse transcription kit (Qiagen, Hilden, Germany) was used to generate cDNA according to the manufacturer’s instructions. Two different protocols were used to generate the cDNA; one with Oligo dT primers and the other with random primers (Promega). PCR was then performed to detect the beta-globin and the MMTV-*env* like transcripts according to the above mentioned protocols.

### Sequencing

The MMTV-*env* like amplified PCR product was excised from the agarose gel. The DNA was purified using Gel extraction kit (Qiagen, Hilden, Germany) and sequenced using the BigDye terminator sequencing and purification kits (Thermo Fisher Scientific, MA, USA) and the genetic analyzer 3500 (Thermo Fisher Scientific, MA, USA).

## Results

**Fig. 3:**
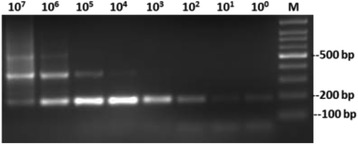
Sensitivity of the newly designed primers for the detection of MMTV-*env* like sequences. A serial dilution of the positive control was used as template for the PCR reaction using MMTV F&R primers. Amplicon size is 187 bp. The numbers indicate the calculated copy number of the positive control fragment per microliter. Four microliters were used per reaction. Detection level is 250 copies per ml. (M) 100 bp DNA ladder

**Fig. 4:**
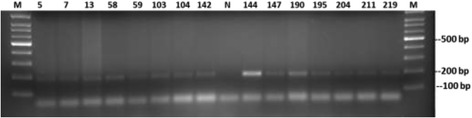
Samples positive for MMTV-*env* like sequences. A new DNA extraction was performed from all samples that gave a positive PCR result. The PCR reaction was repeated to confirm the results. (N) Stands for negative control

The quality of extracted DNA was good as demonstrated by the detection of the β-globin gene in all samples. To detect the MMTV *env* like sequences we designed a new set of primers (MMTV-F and MMTV-R) based on an alignment of the human mammary tumor virus (HMTV) sequences from the PubMed database (Fig. 2). The optimum conditions for these primers were first standardized on a serial dilution of the positive control (kindly provided by professor Pogo) (Fig. 3). All samples were then tested using the new primers. Out of 194 samples, fifteen tissue samples (7.7%) gave positive results (Fig. 4), six (6/101, 5.9%) were from cancerous tissues and nine (9/93, 9.7%) were from non-cancerous surrounding tissues. None of the 51 breast tissues samples from non-breast cancer patients gave positive PCR results.

Out of the 103 breast cancer patients tested, 9 patients (8.7%) showed evidence of MMTV like *env* proviral sequences in their breast tissue (Table 1). Six patients (5.8%) showed MMTV like sequences in both breast cancer tissues and non-cancerous surrounding tissues, none of the patients had MMTV positivity only in cancer tissues and 3 patients (2.9%) showed positivity only in non-cancerous surrounding tissues but not in cancer tissues (Table 1). The prevalence of MMTV in Saudi breast cancer patients alone was 5.97%. Table 2 shows the prevalence of MMTV in breast cancer from different nationalities in the study.

**Table 1:** Frequency of MMTV *env* sequence in relation to age of patient, grade of cancer and lymph node metastasis

Variables	frequency of MMTV *env* positivity in both tumor and normal tissue (*n* = 6/103) 5.8%	frequency of MMTV env positivity in tumor (*n* = 0/103) 0.0%	frequency of MMTV env positivity in normal tissue (*n* = 3/103) 2.9%	Total (*n* = 9/103) 8.7%
Age (years)				
20–29	0	0	0	0
30–39	1 (16.7%)	0	1 (33.3%)	2 (22.2%)
40–49	1 (16.7%)	0	2 (66.7%)	3 (33.3%)
50–59	3(50.0%)	0	0	3 (33.3%)
60–69	0	0	0	0
70–79	1 (16.7%)	0	0	1 (11.1%)
*P*-value	0.35^a^	-(None reported as the observed positivity is zero)	0.50^a^	0.45^a^
Grade				
1	0	0	1 (33.3%)	1 (11.1%)
2	2 (33.3%)	0	2 (66.7%)	4 (44.4%)
3	4 (66.7%)	0	0	4 (44.4%)
*p*-value	0.69^a^	**–**	0.34^a^	0.91^a^
Presence of lymph nodes metastasis				
No	6(100.0%)	0	2 (66.7)	8 (88.9%)
Yes	0	0	1(33.3%)	1 (11.1%)
*p*-value	0.003^a^	**–**	0.60^a^	0.002^a^

^a^Fisher’s exact due to small numbers

**Table 2:** MMTV positivity in the study population of different nationalities

Nationality	Number of patients	MMTV positive patients/number of patients (%)	Region	MMTV positive patients/number of patients (%)
Pakistani	1	0/1 (0)	Asian	1/11 (9.1%)
Indian	4	0/4 (0)		
Philippines	5	0/5 (0)		
Sri Lankan	1	1/1 (100)		
Lebanese	1	0/1 (0)	Middle East	6/77 (7.8%)
Bahraini	1	1/1 (100)		
Palestinian	2	0/2 (0)		
Jordanian	2	1/2 (50)		
Saudi	67	4/67 (5.97)		
Syrian	1	0/1 (0)		
Yemini	3	0/3 (0)		
Somali	1	0/1 (0)	Africa	2/15 (13.3%)
Sudanese	5	1/5 (20)		
Egyptian	8	0/8 (0)		
Moroccan	1	1/1 (100)		
Total	103	9/103 (8.7)		9/103 (8.7)

Despite the use of two different primers, oligo dT and random primers, for the generation of the total cDNA, neither MMTV-*env* RNA sequence nor β-globin gene transcripts could be detected in any of the samples.

**Fig. 5:**
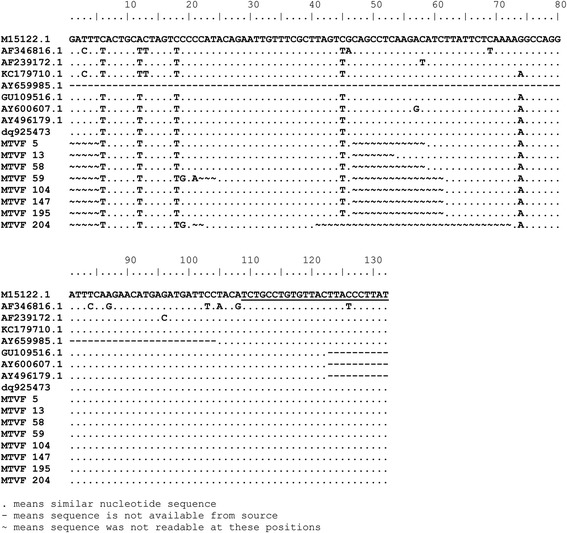
Alignment of the detected MMTV-like *env* sequences (MTVF5, 13, 58, 59,104,147,195,204) shows very high similarity to HMTV

Sequencing the MMTV-*env* like PCR product, from eight of the positive samples, showed that it resembles the human mammary tumor virus (HMTV) sequences of the following accession numbers GU109516.1, AY496179.1, and dq925473 with 100% similarity in the sequenced regions (Fig. 5).

## Discussion

Detection of MMTV *env* sequences is variable in different parts of the world ranging from 0.8–74% (Table 3). The reason for this geographical variation in addition to the role played by this virus in breast cancer and the mode of transmission are not completely understood. However, there is accumulating evidence supporting the association of this virus with the risk of breast cancer. In fact, Wang et al. performed a systematic search of multiple databases to explore this association and found that the presence of MMTV like virus is associated with increased risk of developing breast cancer and is significantly higher in western countries than in Asian countries [21]. Additionally, MMTV-like sequences of the *env* gene were found in breast biopsies of Australian females before and after developing breast cancer, a criterion which fulfills a possible causal correlation between the virus and breast cancer [22]. MMTV seems to have jumped into human population in old times through zoonotic transmission from mice and maintained itself using certain routes of transmission. Two routes of transmission, saliva and milk, were documented in literature. MMTV DNA was detected in saliva of breast cancer patients, healthy adults and children but not in newborns [23]. The same study reported the detection of MMTV RNA in saliva of breast cancer patients and their salivary glands suggesting saliva as a route of transmission among humans [23]. MMTV sequences were also detected in the milk of healthy lactating women [24, 25] and lactating women at high risk of developing breast cancer [25] suggesting another route of MMTV transmission among humans.

**Table 3:** Prevalence of MMTV like *env* sequence in human breast cancer samples and controls in published articles between 1995 and 2014 from different geographical location

Year	Geographic location	Breast cancer samples		Control samples		Reference
		Number	MMTV-like *env* positivity no. (%)	Number	MMTV-like *env* positivity no. (%)	
1995	USA	314	121 (38.5%)	136 (29 fibroadenoma and 107 reduction mammoplasty)	4 (2.9%)	[5]
2000	USA	72	27 (37%)	35 (25 negative biopsy, 5 reduction mammoplasty and 5 adjacent breast tissue)	0 (0%)	[7]
2001	USA	106	32 (30.1%)	106 (paired breast tissue)	1 (0.94)	[6]
2003	Australia	45 Caucasian Australian, 120, Vietnamese 41 Vietnamese- Australian	19 (42.2%) Caucasian Australian, 1(0.8%), Vietnamese 0 (0%) Vietnamese- Australian	111 Australian and 60 Vietnamese (normal breast tissues)	2 (1.8%) Australian and 0 (0%) Vietnamese	[17]
2004	Australia	128	50 (40%)	–	–	[43]
2004	Australia	136 + 13 male breast cancer	43 (32%) + 8 (62%)	164 (111 normal breast tissue,25 fibroadenoma, 4 hyperplasia, 25 fibrocystic) + 52 male (gynecomastia)	15 (9%) + 10 (19%)	[44]
2004	Tunisia	38	28 (73%)	–	–	[38]
2006	Italy	45	15 (33%)	40 (8 normal breast tissue, 4 blood from breast cancer patients, 20 other cancers and 8 blood from healthy blood donor)	0	[39]
2007	Mexico	119	5 (4.2%)	–	–	[45]
2008	Australia	50	28 (56%)	–	–	[10]
2008	Tunisia	122	17 (13.9%)	122 (adjacent breast tissue)	0 (0%)	[34]
2008	Japan	46	0 (0%)	3 benign breast tumor	0 (0%)	[35]
2010	Australia	74	33 (45%)	29 (reduction mammoplasty)	0 (0%)	[37]
2011	Australia	42	0 (0%)	–	–	[46]
2012	Australia	50	39 (78%)	40 (milk from normal lactating women)	8 (32%)	[9]
2012	Iran	50+ 300 blood from breast cancer patient	0 (0%)	300 (blood from healthy donor)	0 (0%)	[36]
2013	Mexico	86	0 (0%)	65 (adjacent breast tissue)	0 (0%)	[47]
2013	Iran	40	0 (0%)	–	–	[48]
2014	Mexico	458	57 (12.4%)	458 (adjacent breast tissue)	72 (15.7%)	[33]
2014	Pakistan	80+ 80 paired blood samples	16 (20%)	–	–	[40]
2014	Morocco	42	24 (57%)	18 (matched normal breast tissue from safety margins)	6 (33.3%)	[41]
2017	Pakistan	250 samples	29.3%	–	–	[49]
2017	Myanmar	58 samples	1 (1.7%)	–	–	[50]

Interestingly, MMTV pathogenesis in human extends to other diseases. MMTV sequences were detected in liver disease biopsies [26] and was found to be associated with primary biliary cirrhosis [27, 28], autoimmune and idiopathic liver diseases [29]. The association of MMTV with primary biliary cirrhosis was not supported by all studies [30]. However, the mitigation of cholangitis using combination antiretroviral therapy in a mouse model of liver disease support the notion of a role for retrovirus in development of liver disease [28].

Breast cancer in Saudi Arabia, like in other parts of the world, constitutes a major health problem. According to the Saudi Arabian Cancer Incidence Report of 2010, breast cancer accounts for 15% of all cancers among Saudis of all ages and 27.4% of cancers in females. Furthermore, Ibrahim et al. found that breast cancer in Saudi women of the eastern province is characterized by earlier onset and more malignant behavior compared to women in the USA with median age of less than 50 years [19]. Since young age has been identified as an independent risk factor for breast cancer relapse and poor prognosis [31, 32] studies exploring the behavior of breast cancer in Saudi women, its prognostic factors and its pathophysiology are urgently needed.

This study reports, to the best of our knowledge, for the first time the detection of MMTV *env* sequences in breast cancer tissues from breast cancer patients in Saudi Arabia and in the Arab patients from the Gulf area at all. The prevalence of MMTV like *env* proviral sequences was 8.7% in breast cancer patients in total and 5.97% (4/67) in Saudi patients (Table 2) but the virus was not detectable in breast tissue from non-breast cancer individuals. The small number of non-Saudi patients in the study precluded a firm conclusion about the prevalence of MMTV in females from other nationalities (Table 2). However, clustering patients according to the region of origin suggested that Africa might have higher frequency of MMTV in breast cancer. A separate study with a lager sample size is required to confirm this hypothesis (Table 2). These findings are in concordance with the low prevalence reported in other countries like Mexico [33], Tunisia [34], Japan [35], Australia [17], and Iran [36] (Table 3). However, the prevalence of MMTV positivity in Saudi Arabia is lower than that detected in other studies from Australia [9, 10, 37] Tunisia [38], USA [5–7], Italy [39], Pakistan [40] and Morocco [41] (Table 3).

MMTV *env*-like sequences were detected simultaneously from cancerous and normal tissue from the same patient in most of the positive cases. However, in 2.9% of the patients, MMTV *env*-like sequences were detected in normal tissue only. Similar finding was reported from other studies previously [33, 41]. Detection of the *env*-like sequences from tumor-adjacent normal tissue could indicates that these cells were not yet transformed by the virus and more distant tissue should be used as a control. It could also bring back the discussion about the endogenous origin of the virus. Nonetheless, detection of the virus only in normal tissue supports the notion of the exogenous origin of the virus and that it was not inherited through the germline. Moreover, it proposes a post tumor infection scenario and excludes a role for the virus in formation of the tumor in these patients. We have never worked previously with MMTV or had any of the other laboratories in our institution excluding the possibility of contamination. Additionally, we have strictly worked in discrete areas and the positive control was added separately in another room. Furthermore and to confirm the authenticity of the PCR results, a second DNA extraction and PCR was made for all samples that gave positive results with the first screen (Fig. 4). Furthermore, MMTV proviral sequences were not detected in breast tissue samples collected from patients with no breast cancer.

There was no significant association between MMTV positivity and patients age or grade of tumor, but there was significant association between MMTV positivity in both cancerous and normal adjacent tissue and absence of lymph node metastasis (*p* value 0.002) (Table.1). Nevertheless, MMTV positivity was higher in patients with more advanced disease (grade 3) in comparison to grade 1 and 2 tumor.

This study adds to the accumulating evidence of the association of MMTV like viruses and human breast cancer particularly advanced tumor and rules out the presence of a significant association between the viral presence and age of the patients and grade of the tumor. At the same time, the low percentage of MMTV positivity (8.7%) found in this study could not alone explain the pattern of breast cancer in this area and other studies are needed to further explore the role of the MMTV in breast cancer. Interestingly, a recent study showed that priming mice with the MMTV-p14 signal peptide elicits a protective immune response against MMTV induced tumor and the use of anti-p14 antibodies or the T-cells from p14-immunized mice is an effective therapeutic intervention [42]. This study provides a promising approach for prevention and treatment of MMTV-associated human tumors.

## Conclusion

Using a newly designed PCR based on sequence alignment of available MMTV *env* sequences, this study detected for the first time in the Arab Gulf region the presence of MMTV *env* sequences in 9/103 breast cancer patients (8.7%). In addition, the detection of the sequences in adjacent normal tissues alone in 3 patients (2.9%) support the hypothesis of the exogenous origin of the virus and therefore provide a baseline for further studies on viral transmission, infectivity and pathogenesis which are highly needed to further understand the role of MMTV in human breast cancer.

## Abbreviations

FFPE: Formalin fixed paraffin embedded
HERV: Human Endogenous Retrovirus
HMTV: Human Mammary Tumor Virus
MMTV *env*: Mouse Mammary Tumor Virus envelope gene
MMTV: Mouse Mammary Tumor Virus
PCR: polymerase chain reaction
SCRELC: Standard Committee for Research Ethics on Living Creatures
